# The Flagellum of *Pseudomonas aeruginosa* Is Required for Resistance to Clearance by Surfactant Protein A

**DOI:** 10.1371/journal.pone.0000564

**Published:** 2007-06-27

**Authors:** Shiping Zhang, Francis X. McCormack, Roger C. Levesque, George A. O'Toole, Gee W. Lau

**Affiliations:** 1 Division of Pulmonary and Critical Care Medicine, University of Cincinnati College of Medicine, Cincinnati, Ohio, United States of America; 2 Centre de Recherche sur la Fonction Structure et Ingenierie des Proteines, Pavillon Charles-Eugene Marchand et Faculte de Medecine, Universite Laval, Ste-Foy, Quebec, Canada; 3 Department of Microbiology and Immunology, Dartmouth Medical School, Hanover, New Hampshire, United States of America; 4 Department of Pathobiology, University of Illinois at Urbana-Champaign, Urbana, Illinois, United States of America; Massachusetts General Hospital and Harvard Medical School, United States of America

## Abstract

Surfactant protein A (SP-A) is an important lung innate immune protein that kills microbial pathogens by opsonization and membrane permeabilization. We investigated the basis of SP-A-mediated pulmonary clearance of *Pseudomonas aeruginosa* using genetically-engineered SP-A mice and a library of signature-tagged *P. aeruginosa* mutants. A mutant with an insertion into *flgE,* the gene that encodes flagellar hook protein, was preferentially cleared by the SP-A^+/+^ mice, but survived in the SP-A^−/−^ mice. Opsonization by SP-A did not play a role in *flgE* clearance. However, exposure to SP-A directly permeabilized and killed the *flgE* mutant, but not the wild-type parental strain. *P. aeruginosa* strains with mutation in other flagellar genes, as well as mucoid, nonmotile isolates from cystic fibrosis patients, were also permeabilized by SP-A. Provision of the wild-type *fliC* gene restored the resistance to SP-A-mediated membrane permeabilization in the *fliC*-deficient bacteria. In addition, non-mucoid, motile revertants of CF isolates reacquired resistance to SP-A-mediated membrane permeability. Resistance to SP-A was dependent on the presence of an intact flagellar structure, and independent of flagellar-dependent motility. We provide evidence that flagellar-deficient mutants harbor inadequate amounts of LPS required to resist membrane permeabilization by SP-A and cellular lysis by detergent targeting bacterial outer membranes. Thus, the flagellum of *P. aeruginosa* plays an indirect but important role resisting SP-A-mediated clearance and membrane permeabilization.

## Introduction

*Pseudomonas aeruginosa* is a ubiquitous Gram-negative pathogen and a major cause of morbidity in patients with nosocomial pneumonia and cystic fibrosis (CF). Environmental strains and most clinical isolates of *P. aeruginosa* involved in acute infections are flagellated and motile [1]–[3]. In contrast, approximately 39% of *P. aeruginosa* isolated from chronically infected CF patients are nonmotile [4], [5]. Other studies have suggested that exposure to airway surface liquid from CF patients quickly represses the expression of *P. aeruginosa* flagellin, the major structural protein of bacterial flagella [6]. This repression is mediated by the AlgT-dependent transcriptional regulator AmrZ [7]. The *P. aeruginosa* flagellum is an important virulence factor [3] and a potent activator of host immunity [8]. A number of studies have shown that *P. aeruginosa* strains lacking flagella are much less readily phagocytosed by alveolar macrophages and polymorphonuclear leukocytes [4], [5]. Thus, the loss of flagellar expression within the CF airway may represent an adaptive response that allows *P. aeruginosa* to evade detection and clearance by host defense mechanisms during the chronic phase of CF lung infection [9].

SP-A is a large oligomeric protein composed of 18 subunits, each containing a collagen-like sequence and a calcium-dependent carbohydrate recognition domain, that is intimately associated with surfactant phospholipids lining the air-liquid interface of the lung [10], [11]. Upon encountering an inhaled bacterial pathogen, SP-A dissociates from the phospholipid membrane and binds to the microbe by calcium dependent ligation of surface bacterial glycoconjugates, including lipopolysaccharide. The association of SP-A with the bacteria enhances uptake by phagocytes, and modulates inflammatory responses including cytokine production and the oxidant burst [11]–[14]. Recent data from our group indicates that SP-A also directly inhibits the growth of gram-negative bacteria such as *Escherichia coli, Bordetella pertussis*, and *P. aeruginosa* by permeabilizing the bacterial membrane [15]–[17]. In general, rough bacterial mutants decorated with truncated LPS species were more readily permeabilized by SP-A than smooth strains [15]–[17], and incorporation of rough but not smooth LPS multilamellar liposomes composed of bacterial phospholipids conferred susceptibility to SP-A-mediated permeabilization [18].

We recently adapted signature tagged mutagenesis (STM) to comparatively identify microbial factors that confer resistance or susceptibility to SP-A [17]. Subsets of a *P. aeruginosa* library composed of 6912 mutants containing individual gene insertions were instilled into SP-A^+/+^ and SP-A^−/−^ mice. Mutants which are cleared in an SP-A-dependent manner were tested for susceptibility to SP-A-mediated opsonization, phagocytic killing and permeabilization. In this paper, we characterize a SP-A sensitive *P. aeruginosa* mutant identified by comparative STM, the flagellar mutant *flgE*.

## Results

### The P. aeruginosa Flagellar Gene flgE Confers Resistance to SP-A

**Figure 1:**
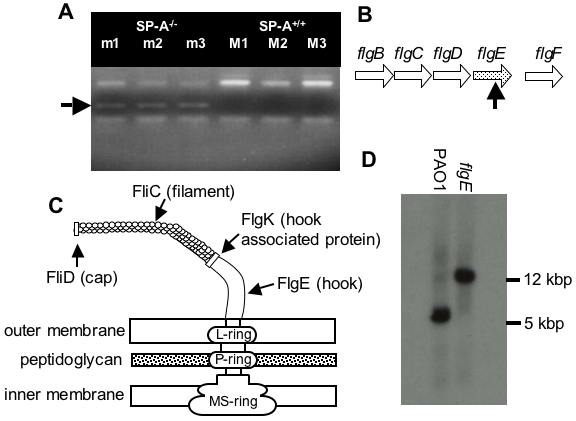
The Flagellar Mutant *flgE* Is Preferentially Cleared from the Lungs of SP-A^+/+^ Mice. (A) Pools of 72 uniquely-tagged mutants were intranasally inoculated into three SP-A^+/+^ (M1, M2, M3) and three SP-A^−/−^ (m1, m2, m3) mice. Mouse lungs were harvested, homogenized and plated. Bacteria colonies were collected for genomic DNA extraction. PCR-amplification of tags was performed to screen for the presence or absence of each of the 72 mutants. The band indicative of the *flgE* mutant was present in the SP-A^−/−^ pool, but absent in the SP-A^+/+^ pool. (B) Genetic organization of the *flg* operon in *P. aeruginosa*. The horizontal arrows (*flgB, flgC, flgD, flgE, flgF*) represent transcriptional directions of the genes coding for flagella proteins. The vertical black arrow indicates the approximate insertion site within the mutated open reading frame of *flgE*. (C) A schematic representation of flagellar structure. FliC is flagellar filament protein, FliD is flagellar cap protein, FlgE is flagellar hook protein, and FlgK is flagellar hook associated protein. (D) Restriction fragment length polymorphism analysis between wild-type PA01 and *flgE* mutant indicates that the *flgE* mutation was caused by transposon insertion into the cloned gene.

We utilized a *P. aeruginosa* PA01 STM mutant library [17], [19] and a SP-A deficient mouse model to identify putative microbial targets for SP-A. We identified an STM mutant that was able to grow in the lungs of SP-A^−/−^ but not SP-A^+/+^ mice, as indicated by the presence of a PCR amplified oligo tag in bacterial output from SP-A^−/−^ mouse lungs, and the absence of a PCR amplified oligo tag in bacterial output from SP-A^+/+^ mouse lungs (Figure 1A).

The disrupted genetic locus was cloned by plasmid rescue and sequenced. The DNA sequences flanking the transposon, pUTminiTn*5*Km2 (Table 1), were analyzed by comparison with online databases (www.pseudomonas.com), and revealed a transposon insertion into the *flgE* (*PA1080*) gene, (Figure 1B), between nucleotide 1021 and 1022. Because the *flgE* gene is the final open reading frame (ORF) within the operon that encodes four genes, *flgB, flgC, flgC* and *flgD*, no polarity effect is expected. The *flgE* gene encodes the hook structure on the flagellum from which the flagellar filament protrudes (Figure 1C) [20]. The *flgE* mutant was determined to harbor a single transposon insertion after hybridization with the transposon vector pUTminiTn*5*Km2 (Table 1) (data not shown). A DNA fragment that flanked both ends of the transposon integration in the *flgE* gene was cloned from the wild-type PA01. A radiolabeled-probe that was synthesized from the flanking DNA fragment hybridized to a higher molecular weight *Pst* I restricted DNA fragment in the chromosome of the *flgE* mutant than the parent strain PA01 (Figure 1D). These results confirmed that the transposon inserted singly into the *flgE* gene.

**Table 1:** Bacterial Strains and Plasmids Used

Bacterial Strains	Relevant characteristics	Reference
*P. aeruginosa*		
PA01	Wild-type	46
*PA01-gfp*	PA01 harboring pUCP19-*gfp*	17
*flgE*	PA01 miniTn5Km2*flgE*, flagellar mutant	This study
PA01	Wild-type	M. Vasil [47]
PAO-fliC	PAO1 *fliC*::Gm^r^	27
PAOC-fliC	PAOC-complemented on the chromosome with the PA01 *fliC* gene at the *attB* site	49
PAO-fliD	PAO1 *fliD*::Gm^r^	26
PA14	Wild-type	48
Δ*motAB*	*motAB* deletion strain of PA14	29
Δ*motCD*	*motCD* deletion strain of PA14	29
Δ*motAB* Δ*motCD*	Both sets of *mot* genes, *motAB* and *motCD* deleted from PA14	29
*flgK*	PA14 *flgK*::Tn*5*(Tc^r^)	32
CF 51	mucoid CF isolate	This study
CF51N	nonmucoid revertant of CF51	This study
CF90	mucoid CF isolate	This study
CF90N	nonmucoid revertant of CF90	This study
Plasmids		
pUTminiTn5Km2	R6K-based suicide delivery plasmid, Km^r^	19
pUCP19-gfp	polylinker *lacZ*, lac^iq^ selection, *bla, gfp*	17
PVL1393	*bla*	Invitrogen (Carlsbad, CA)

### The SP-A-sensitive STM Mutant, *flgE*, Competes Poorly With the Parental PA01 Strain in the Lungs of SP-A^+/+^ Mice

**Figure 2:**
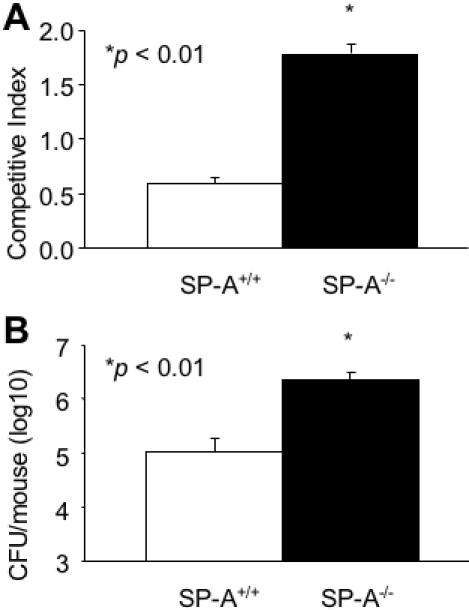
The *flgE* Mutant Is Attenuated in SP-A^+/+^ Mice. (A) *In vivo* competition between the PA01 and *flgE* was assessed after intranasal pulmonary inoculation of 6-week old SP-A^+/+^ and SP-A^−/−^ mice. The mean competitive index (CI) was calculated from bacterial colonies recovered 16 hr after infection. The competitive index (CI) is defined as the output ratio of mutant to wild-type bacteria divided by the input ratio of mutant to wild-type bacteria. Data are the mean±SE, *n* = 4. *p<0.05. (B) Single respiratory tract infection of *flgE* mutant bacteria was performed by intranasal inoculation in SP-A^+/+^ and SP-A^−/−^ mice. CFU were counted in lung homogenates 16 hr after inoculation. Data are the mean CFU±SE, n = 5. *p<0.01.

Apart from confronting pulmonary host defenses, the STM screening strategy used requires individual mutants to compete with 71 other STM mutants within individual library pools. Competitive mixed infection assays have been used to assess the fitness of individual mutants versus their parental strains during *in vivo* infection [17], [21]. When inoculated with equal numbers of with-type PA01 into the lungs of mice, the *flgE* mutant was only 33% as competitive as PA01 in SP-A^+/+^ animals (Figure 2A). In contrast, the *flgE* mutant competed comparably, if not better than, the wild-type PA01 in SP-A^−/−^ mice (Figure 2A). We also tested the *flgE* mutant in single infection studies. Eighteen hours after intranasal inoculation, the viable bacterial counts of *flgE* mutant bacteria were 1.3 log higher in lung homogenates from SP-A^−/−^ mice compared to SP-A^+/+^ mice (Figure 2B). Our results indicate that flagellar function plays an important role against SP-A-mediated clearance of *P. aeruginosa*.

### Opsonization by SP-A Does Not Play A Role in the Preferential Clearance of the *flgE* Mutant *In Vivo*

**Figure 3:**
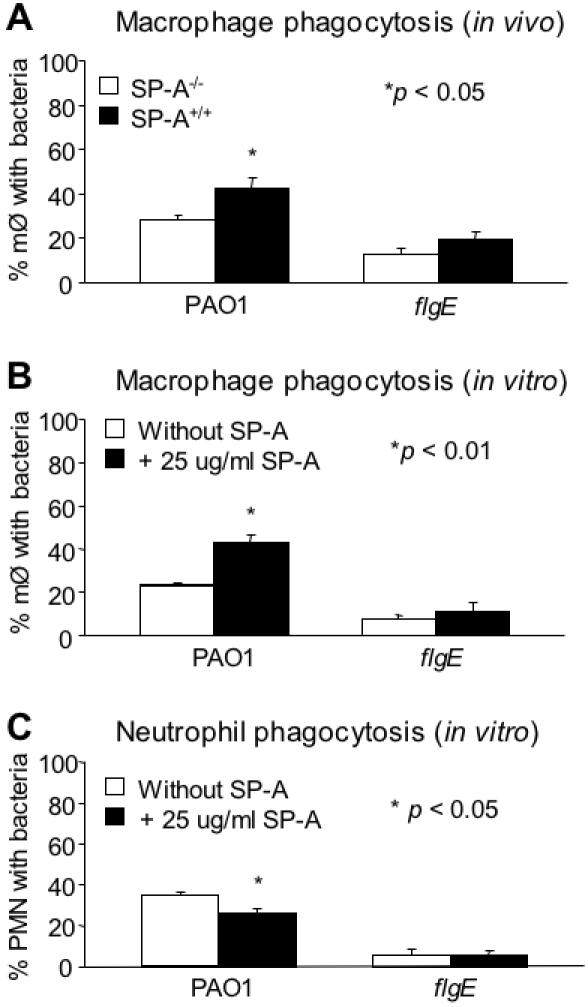
SP-A-Mediated Opsonization Does Not Play A Critical Role in the Preferential Clearance of *flgE* Bacteria. (A) Analysis of *in vivo* phagocytosis was performed by intranasally inoculating live, GFP-expressing PA01 and *flgE* bacteria into SP-A^+/+^ and SP-A^−/−^ mice (n = 3). Macrophages were recovered by bronchoalveolar lavage 2 hr later. The percentage of macrophages with internalized bacteria was determined using a phase contrast fluorescence microscope. Two hundred macrophages were counted for each mouse. Data are the mean±SE, *n* = 3. *p<0.05. (B) Analysis of *in vitro* phagocytosis was performed using live, GFP-expressing PA01 and *flgE* bacteria with alveolar macrophages isolated from SP-A^−/− ^mice. The percentage of macrophages with internalized bacteria was determined using a phase contrast fluorescence microscope. Two hundred macrophages were counted for each mouse. Data are the mean±SE, *n* = 3. *p<0.01. (C) *In vitro* phagocytosis of PA01 and *flgE* by human neutrophils isolated from the blood of healthy volunteers. Phagocytosis experiments were performed as described for (A). Two hundred neutrophils were counted. Data are the mean±SE, *n* = 3. *p<0.05. PMN = Neutrophils. M∅ = Macrophages.

Previous studies have demonstrated that flagella act as a major ligand for nonopsonic phagocytosis of *P. aeruginosa*, and are required to trigger internalization [4]–[5]. Consequently, *P. aeruginosa* lacking flagella are resistant to nonopsonic phagocytosis by macrophages and neutrophils. In multiple studies, SP-A has been reported to opsonize pathogens and enhance their phagocytosis by alveolar macrophages and neutrophils [22]–[24]. To rule out SP-A-mediated opsonization contributed to the differential clearance of *flgE* mutant in SP-A mouse models, we compared the phagocytosis of *flgE* to PA01 bacteria in SP-A^+/+^ and SP-A^−/−^ mice after intranasal inoculation *in vivo*, and in the presence and absence of SP-A, *in vitro*. Macrophages with internalized GFP-expressing *flgE* and PA01 were enumerated by phase contrast fluorescence microscopy. Uptake of PA01 bacteria was noted in 42.1±7.6% of alveolar macrophages isolated 2 hr after intranasal inoculation from SP-A^+/+^ mice, compared to 28.5±3.0% of alveolar macrophages with PA01 from SP-A^−/−^ mice (Figure 3A). These results suggest that SP-A facilitates the *in vivo* macrophage-mediated phagocytosis of PA01 bacteria, which have intact flagella. In contrast, the loss of flagella in *flgE* mutant bacteria results in lower total uptake by alveolar macrophages when compared to wild-type PA01 bacteria. Specifically, *flgE* bacteria were internalized in only 19.8±5.7% of alveolar macrophages from SP-A^+/+^ mice compared with 12.7±5.1% from SP-A^−/−^ mice (Figure 3A). Thus, *in vitro*, SP-A enhanced phagocytosis of PA01 by approximately 50% but did not have any effect on the phagocytosis of *flgE* bacteria (Figure 3B). Similarly, as shown in Figure 3C, the uptake of PA01 and the *flgE* mutant by neutrophils were not increased in the presence of SP-A (Fig. 3C). In fact, SP-A-opsonized PA01 was phagocytosed less efficiently than untreated bacteria by neutrophils. However, the uptake of PA01 was significantly greater than *flgE,* irrespective of the presence or absence of SP-A.

Collectively, our results suggest that while flagella enhance internalization of *P. aeruginosa* into macrophages and neutrophils, SP-A-mediated macrophage and neutrophil phagocytosis are not responsible for the preferential clearance of *flgE* mutant bacteria from SP-A^+/+^ mice. These results also confirm data from other studies that flagella serve as critical ligands for the internalization of bacteria [4], [5].

### An Intact Flagellum is Required to Confer Resistance of P. aeruginosa to SP-A-mediated Membrane Permeabilization

**Figure 4:**
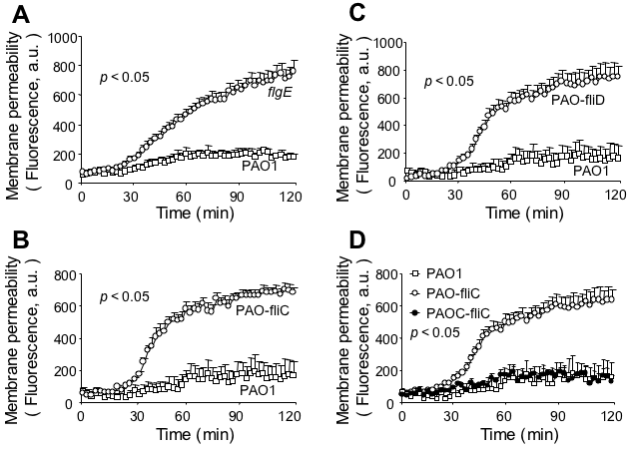
SP-A Preferentially Permeabilizes Flagellum-Deficient Mutants of *P. aeruginosa*. (A–C) SP-A preferentially permeabilizes flagellum-deficient *flgE, fliC and fliD* mutants. Bacterial cells from the wild-type PA01 and mutant strains *flgE, fliC* (PAO-fliC), and *fliD* (PAO-fliD) were exposed to 50 µg/ml human SP-A in the presence of the phosphatase substrate ELF97. Fluorescence was measured for 120 min using a fluorimeter and expressed as arbitrary units (*a.u.*). Data are the mean±SE, *n* = 6–8. The difference in membrane permeabilization between *flgE* mutant and PA01 was significant from 31^st^-min onward. *p<0.05. The differences in membrane permeabilization between PAO-fliC and PA01, and between PAO-fliD and PA01 were both significant from 32^nd^-min onward, respectively. *p<0.05. (D) Genetic complementation of flagellar function restores resistance to SP-A-mediated membrane permeabilization. PAOC-fliC (open square), a *fliC* mutant harboring a copy of wild-type *fliC* gene is as resistant to SP-A-mediated membrane permeabilization as the parental strain PA01 (open circle). Data are the mean±SE, *n* = 6. The differences in membrane permeabilization between *fliC* and PA01, and between *fliC* and PAOC-fliC, were significant from 32^nd^-min, and 34^th^-min onward, respectively. *p<0.05.

We have recently shown that SP-A is capable of directly killing Gram-negative bacteria and fungi by membrane permeabilization and inhibition of macro-molecular synthesis, independent of macrophage-mediated phagocytosis [15], [17], [25]. We examined whether direct membrane permeabilization contributed to the preferential clearance of the flagellar-deficient *flgE* mutant. The *flgE* mutant bacteria were permeabilized by SP-A to an extent that was 3.4 fold greater than the wild-type strain PA01 after exposure to SP-A for 120 minutes (Figure 4A).

We postulate that the susceptibility of the *flgE* mutant to SP-A-mediated membrane permeabilization is due to its inability to assemble a flagellum. We tested additional flagellar mutants, *fliC* (PAO-fliC) and *fliD* (PAO-fliD) [26], [27], for their susceptibility to membrane permeabilization by SP-A. The *fliC* gene encodes flagellin, which comprises the filament of a flagellum whereas the *fliD* gene encodes the cap of a flagellum (Figure 1C). Both PAO-fliC and PAO-fliD mutant bacteria exhibited increased susceptibility to SP-A-mediated membrane permeabilization to an extent that was 3.3 and 4.4 fold greater than the wild-type strain PA01, respectively, at 120 min post-exposure to SP-A (Figures 4, B and C). The levels of PAO-fliC and PAO-fliD bacteria susceptibility were comparable to the *flgE* mutant (Figure 4A), and to the *E. coli* K12 [15]. These results indicate that an intact flagellum or flagellar function is essential for resistance to SP-A-mediated membrane permeabilization, and is consistent with previous finding that flagellum is required for acute lung infection by *P. aeruginosa* [3].

To confirm that the loss of flagella confers susceptibility of *P. aeruginosa* to SP-A, we determined if provision of the wild-type *fliC* gene *in trans* to the *fliC*-deficient mutant bacteria could restore the resistance to SP-A-mediated membrane permeabilization. Importantly, while PAO-fliC mutant bacteria were susceptible to SP-A-mediated membrane permeabilization, the complemented PAOC-fliC bacteria, were as resistant to SP-A-mediated membrane permeabilization as the wild-type PA01 (Figure 4D). These results indicate that an intact flagellar structure or function is important in protecting *P. aeruginosa* from membrane destabilization by SP-A.

### Membrane Permeabilization by SP-A Directly Kills Flagellar Mutants

**Figure 5:**
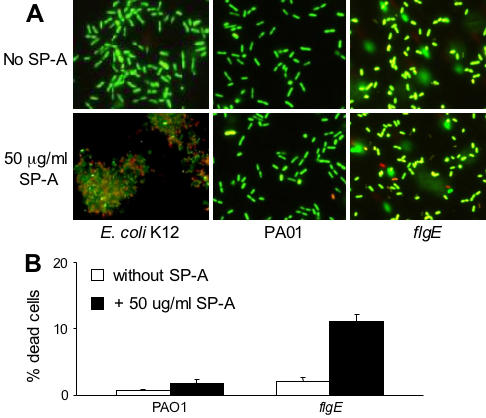
SP-A Directly Kills *flgE* by Membrane Permeabilization. (A) Live/dead staining was performed on *E. coli* K12, PA01 and *flgE* cells following incubation with 50 µg/ml SP-A. Green-stained cells are alive whereas red-stained cells are dead. Robust aggregation of *E. coli K12* but not *P. aeruginosa* strains by SP-A was observed. (B) Enumeration of live or dead bacteria following a 1 h exposure to hSP-A. At least 1,000 bacterial cells were counted under a fluorescence microscope. The mean±SD of two experiments is shown.

We next determined if SP-A-mediated membrane permeabilization kills flagellar mutants. Live/dead staining, based on exclusion of propidium iodide from the live cells, was immediately performed on bacterial cells following 120 min of exposure to SP-A. Viable cells are stained green while dead cells are stained red. As shown in Figure 5A, SP-A did not kill wild-type PA01 (green stained). In contrast, mixtures of green- and red-stained cells of *flgE* were visible following treatment with SP-A. Approximately 11% of the *flgE* bacteria were killed within 60 minutes of exposure to SP-A. Bacterial aggregation can affect viability. However, in contrast to the robust SP-A induced aggregation of *E. coli* K12, SP-A did not aggregate PA01 or *flgE* bacteria (Figure 5B). These results suggest that the loss of flagella render the *P. aeruginosa* vulnerable to membrane permeabilization and killing by SP-A. Furthermore, aggregation of *P. aeruginosa* is unlikely to play a role in the preferential clearance of the flagellar mutants.

### Mucoid, Nonmotile P. aeruginosa Isolated from CF Patients are Susceptible to SP-A-mediated Membrane Permeabilization

**Figure 6:**
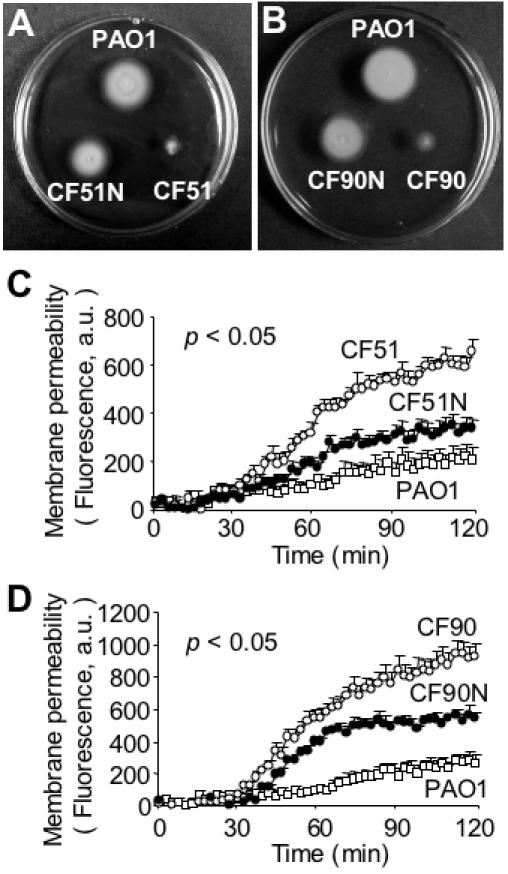
Nonmucoid Motile Revertants of Clinical CF *P. aeruginosa* Strains Are More Resistant to SP-A-Mediated Membrane Permeabilization Than Their Parental Isolates. (A–B) The mucoid clinical CF *P. aeruginosa* strains CF51 and CF90 were nonmotile, whereas their nonmucoid motile revertants CF51N and CF90N had regained their flagellar-based motility on swimming motility agar plates. The wild-type strain PA01was used as control for motility assay. (C–D) Both CF51N and CF90N were more resistant to SP-A-mediated membrane permeabilization than their parental isolates, CF51 and CF90, respectively. Data are the mean±SE, *n* = 8 for each bacterial strain. The differences in membrane permeabilization between CF51 versus PA01; CF51 versus CF51N; and CF51N versus PA01 were significant from 43^rd^-min, 53^rd^-min and 53^rd^-min onward, respectively. *p<0.05. The difference in membrane permeabilization between CF90 versus PA01; CF90 versus CF90N; and CF90N versus PA01 was significant from 38^th^-min, 28^th^-min, and 43^rd^-min onward, respectively. *p<0.05.

As discussed earlier, significant numbers (39%) of *P. aeruginosa* strains isolated from chronically-infected CF patients are both mucoid and nonmotile [4]. The loss of motility is due, in part, to repression of flagellar biosynthesis by the AlgT-dependent transcriptional regulator AmrZ [7] induced by the CF airway fluids [6]. However, mucoid, nonmotile CF *P. aeruginosa* isolates frequently revert to nonmucoid, motile phenotypes when they are grown in static culture under laboratory conditions [28]. We examined the effect of SP-A on two mucoid, nonflagellated strains of *P. aeruginosa* isolated from CF patients. Strains CF51 and CF90 were nonmotile (Figure 6, A and B), and membrane permeabilized by SP-A to an extent that was 3.2 fold and 3.6 fold greater than the wild-type strain PA01, respectively, after 120 min exposure (Figure 6, C and D). The levels of membrane permeabilization in these two CF strains were similar to flagellum-deficient *flgE, fliC* and *fliD* mutants. The overall correlation of flagellar motility and resistance to membrane permeability are summarized in Table 2.

**Table 2:** Correlation of Flagellum Production, Motility and SP-A-Mediated Membrane Permeabilization

Bacterial strains	Flagellum	Motility	Membrane permeabilization*
PA01 (Wild-type)	+	+++	-
*flgE*	-	-	4.3
*fliC*	-	-	4.1
*fliD*	-	-	4.5
PAOC-fliC	+	+++	-
PA14 (Wild-type)	+	+++	-
*flgK*	-	-	7.9
Δ*motAB*	+	+	-
Δ*motCD*	+	++	-
Δ*motAB* Δ*motCD*	+	-	-
CF51	-	-	3.2
CF51N	+	++	1.6
CF90	-	-	3.8
CF90N	+	+	2.4

* Flagellum production was determined by motility assay. Extent of motility was assessed by examining the radius of opacity as a result of bacterial swimming away from the inoculation point. Permeability was determined by measuring the ELF97 fluorescence at excitation and emission wavelengths of 355 and 535 nm.

### Nonmucoid, Motile Revertants of CF *P. aeruginosa* Isolates Reacquire Resistance to SP-A-mediated Membrane Permeabilization

If *P. aeruginosa* flagella play critical roles in resistance to SP-A-mediated membrane permeability, we predicted that nonmucoid, motile revertants of CF isolates CF51 and CF90, named CF51N and CF90N, would have increased resistance to SP-A-mediated membrane permeabilization. Both revertants were nonmucoid, and determined to be motile by swimming motility assay (Figure 6, A and B). The levels of resistance to SP-A-mediated membrane permeabilization were increased by 2.0 and 1.7 fold in CF51N and CF90N, respectively, when compared to parental strains CF51 and CF90 (Figure 6, C and D; Table 2). Collectively, these results confirm that *P. aeruginosa* flagellum plays a crucial role in conferring resistance to SP-A-mediated membrane permeabilization.

### Susceptibility to SP-A-mediated Membrane Permeabilization is Dependent On An Intact Flagellum Structure but Independent of Flagellar-mediated Swimming Motility

**Figure 7:**
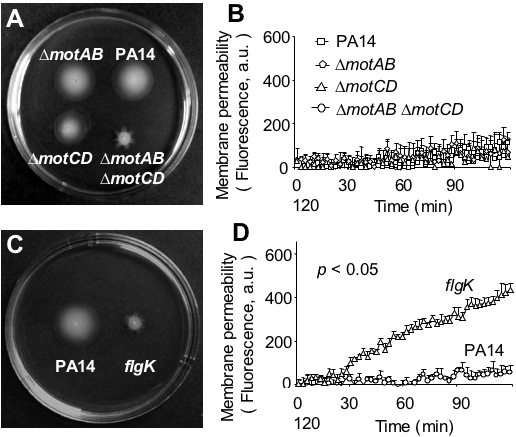
Susceptibility to SP-A-Mediated Membrane Permeabilization Is Dependent on Intact Flagellum Structure but Independent of Flagellar-Mediated Swimming Motility. (A) Wild-type *P. aeruginosa* strain PA14 and its isogenic derivative δ*motAB*, δ*motCD* and δ*motAB*, δ*motCD* were tested for swimming motility and susceptibility to SP-A-mediate membrane permeabilization. Mutant strains δ*motAB* and δ*motCD* were motile, but δ*motAB*, δ*motCD* was nonmotile on swimming motility agar plate. (B) All δ*motAB*, δ*motCD* and δ*motAB*, δ*motCD* mutant strains were resistant to SP-A-mediated membrane permeabilization, indicating that susceptibility to SP-A is independent of flagellar-mediated motility. (C) The *flgK* mutant, which could not assemble flagellum, was nonmotile. (D) The *flgK* mutant was susceptible to SP-A-mediated membrane permeabilization. Data were the mean±SE, *n* = 8 for each bacterial strain. The difference in membrane permeabilization between *flgK* and PA14 was significant from 31^st^-min onward. *p<0.05.

The susceptibility of flagellum-deficient mutants to SP-A could be due to changes in membrane fluidity and rigidity, or due to the loss of motility function. We examined whether the nonmotile *P. aeruginosa* mutants that harbor intact flagella were resistant to membrane permeabilization by SP-A. We utilized *P. aeruginosa* mutants with deletions in *motAB, motCD* or *motAB motCD* genes, all of which encode the stator for flagellum [29]. The stator conducts protons across the inner membrane and couples proton transport to rotation of the flagellar motor [30]. The phenotypic analysis of the deletion mutants demonstrated that the MotAB and MotCD proteins are functionally redundant for swimming (Fig. 7A), as well as for swarming in relatively low agar concentrations [29]. However, under higher agar concentrations, the *motCD* mutant is unable to swarm [29]. The double mutant δ*motAB* δ*motCD* can neither swim (Figure 7A) nor swarm under any conditions tested, but still makes a flagellum [29]. Importantly, δ*motAB*, δ*motCD* and δ*motAB*, δ*motCD* were all as resistant to SP-A-mediated membrane permeabilization as their parental wild-type PA14 (Figure 7B). As a control, we demonstrated that *flgK* mutant, which was derived from PA14, and unable to assemble a flagellum due to the lack of the flagellar hook-associated protein, was nonmotile (Figure 7C) and membrane permeabilized by SP-A to an extent that was 7.9-fold greater than its wild-type parental strain, PA14. These results indicate that resistance to SP-A-mediated membrane permeabilization is dependent on the presence of an intact flagellar structure, rather than the motility function mediated by flagellum.

### The Loss of Flagellum Reduces the Ability of P. aeruginosa to Express Adequate LPS and Destabilize Its Outer Membranes

**Figure 8:**
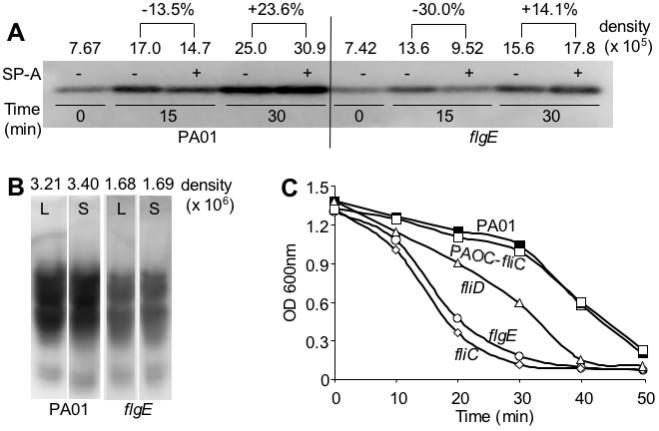
Mutation in the *flgE* Gene Reduces the Ability of *P. aeruginosa* to Synthesize LPS Both in the Presence and in the Absence of SP-A. (A–B) The flgE mutant has reduced ability to synthesize the inner core (A) and B-band-O-antigen (B) of LPS. The induction of LPS biosynthesis was measured using a monocloncal antibody against the inner core sugars and B-band O-antigen of LPS. The fold of increase was quantified against controls using a densitometer. (C) Flagellar mutants are more susceptible to cell lysis by SDS. Wild-type PA01 and the complemented PAOC-fliC strains are more resistant to 0.25% SDS than the flagellum-deficient mutants *flgE, fliC* and *fliD*. Experiments were repeated five times with similar results. The results from one typical experiment are shown.

We have previously shown that LPS is essential for resistance to SP-A-mediated membrane permeabilization in P. aeruginosa [17]. We determined whether the loss of flagellum negatively affected LPS levels on the outer membrane of P. aeruginosa, which could lead to susceptibility to SP-A. As shown in Figure 8A, the amounts of the LPS inner core sugars on P. aeruginosa was dramatically reduced after 15 min exposure to SP-A. However, the reduction is much more severe in the flgE mutant (−30%) when compared to wild-type PA01 (−13.5%). At 30 min post-SP-A exposure, PA01 increased the synthesis of inner core sugars by +23.6% in comparison to +14.1% for flgE. Furthermore, using an antibody against the B-band O-antigen, the flgE mutant was found to harbor 48% and 50% less LPS than the wild-type PA01 during both logarithmic and stationary phases of growth, respectively, in the absence of SP-A (Figure 8B). Collectively, these results suggest that the flagellar-deficient flgE mutant bacteria express lower levels of LPS on its outer membrane.

Gram-negative bacteria with defects in their outer membranes are well known to be more sensitive to lysis by detergents, including sodium dodecyl sulfate (SDS). We examined the flagellar-deficient mutants flgE, fliC and fliD for their increased susceptibility to 0.25% SDS. As shown in Figure 8C, all three mutants were more sensitive to lysis by SDS. In contrast, the wild-type PA01, and PAOC-fliC (complemented fliC mutant) were equally more resistant to SDS. These results support the notion that that the loss of flagellum reduces the expression of LPS and the stability of outer membranes on P. aeruginosa, rendering them susceptible to SP-A-mediated membrane permeabilization and lysis SDS.

## Discussion

In this study, we screened a library of *P. aeruginosa* mutant bacteria in SP-A null and sufficient mice for mutations with increased susceptibility to pulmonary clearance by SP-A. One SP-A sensitive *P. aeruginosa* mutant with a disruption in *flgE*, a gene required for flagellar function, was identified. Enhanced uptake by phagocytes did not play a role in the preferential clearance of *flgE* bacteria, consistent with previous findings that flagellum-deficient mutants are resistant to phagocytosis by macrophages and neutrophils [4], [5]. Rather, *flgE* bacteria were more readily membrane permeabilized and killed by SP-A than its wild-type parental strain, PA01. The importance of flagella in resisting the membrane destabilizing activities of SP-A was verified by multiple independent methods: Firstly, we showed that additional flagellar mutants *fliC, fliD* and *flgK* were also susceptible to SP-A-mediated membrane permeabilization. Secondly, genetic complementation of the *fliC* mutation with a copy of wild-type *fliC* gene restored resistance to SP-A-mediated membrane permeabilization. Thirdly, mucoid, nonmotile CF isolates of *P. aeruginosa* were also membrane permeabilized by SP-A. Finally, motile, nonmucoid revertants derived from the SP-A-susceptible CF isolates were able to reacquire resistance to SP-A. These results reveal that flagellum plays critical roles in conferring resistance to membrane permeabilization by SP-A.

Our genetic analysis suggests that the presence of the flagellar structure rather than the flagellar-dependent motility is largely responsible for the resistance to SP-A-mediated membrane permeabilization. This conclusion is derived from our experimental evidence showing that the δ*motAB*, δ*motCD* mutant, which has a flagellum but is nonmotile [29], is as resistant to SP-A-mediated membrane permeabilization as its isogenic wild-type parental strain, PA14. Thus, it is likely that susceptibility of flagellar-deficient mutants to SP-A-mediated membrane permeabilization is caused by alterations to the outer cell membrane, such as LPS density, charge distribution or phosphohlipid packing density. Several lines of evidence support our prediction: Firstly, we found that exposure to SP-A induced higher levels of LPS in the wild-type *P. aeruginosa* PA01 than its isogenic *flgE* mutant bacteria. Secondly, *flgE* bacteria contain reduced levels of LPS during both logarithmic and stationary phases of growth. Thirdly, flagellar-deficient mutants *flgE, fliC* and *fliD* were all more susceptible to 0.25% SDS, indicative of weakened outer membranes.

Because the nonmotile δ*motAB*, δ*motCD* mutant is resistant to SP-A, the susceptibility to SP-A appears to be independent of flagellar-mediated motility functions. However, we are unable to rule out other flagellum-dependent mechanisms that may protect *P. aeruginosa* from SP-A-mediated membrane permeabilization, including swimming chemotaxis and swarming motility [31], and initiation of biofilm formation through attachment to substrates [32]. Swarming motility is a flagellum-dependent, coordinated, multicellular behavior promoting rapid bacterial colony migration and expansion on more viscous surface, such as the epithelial lining layer that *P. aeruginosa* encounters after inhalation into the alveolar space. In numerous species of Gram-negative bacteria including *P. aeruginosa*, swarmer cells alter their cellular morphology as well as induce the expression of PmrA-B regulated LPS modification genes *pmrHFIJKLMN*, and other LPS biosynthesis genes that are required for successful swarming [33], probably by increasing wettability. Overexpression and modification of LPS contributes to higher levels of resistance to antimicrobial peptides and antibiotics in swarming Gram-nagative bacteria [31], [33]. As such, swarming *P. aeruginosa* cells may be more resistant to SP-A-mediated membrane permeabilization. It is also conceivable that the loss of swimming chemotaxis in the flagellum-deficient mutants render them unable to escape alveolar microenvironments in which SP-A is enriched, such as in surfactant-containing compartments. Another possible explanation, though less likely, is the inability of flagellar mutant to form biofilms. Flagellum-mediate attachment to substrate is the first step in the initiation of biofilm formation [32], a mode of growth which is known to contribute to the resistance of bacteria to antibiotics and other antimicrobial agents [34], [35]. However, biofilms mode of growth is thought to be more important in chronic model of infection, as in the airways of CF patients [9], [36], rather than in the acute pneumonia model of infection.

We found that motile, nonmucoid revertants derived from CF *P. aeruginosa* strains, CF51N and CF90N, were more resistant to SP-A-mediated membrane permeabilization than their mucoid, nonmotile parental isolates CF51 and CF90. This transition correlated with the reacquisition of swimming motility. However, the resistance of CF51N and CF90N to SP-A-mediated membrane permeabilization was not as prominent when compared to the laboratory wild-type strain PA01. There are several possible explanations for this discrepancy. Firstly, it is likely that PA01 and CF51 and CF90 have different levels of intrinsic resistance to SP-A as these are not isogenic strains. Secondly, it is well known that many CF isolates of *P. aeruginosa* harbor mutated forms of LPS. In particular, CF isolates often undergo mutations in genes that participate in the biosynthesis of the highly antigenic B-band O-antigen of LPS [37], which is important for *P. aeruginosa*'s resistance to SP-A [17]. Thus, LPS mutations in the CF51N and CF90N revertants may explain the incomplete restoration of resistance to SP-A-mediated membrane permeabilization even though the flagellar expression is reacquired.

Reduction in the SP-A levels of the alveolar lining fluid, as has been reported in bacterial pneumonia, adult respiratory distress syndrome and CF [38]–[40], may play a role in the susceptibility to pulmonary microbial colonization and infection. Proteases released by neutrophil [41] and *P. aeruginosa* [42], [43] are the most likely causes of SP-A depletion. Thus, the much-reduced SP-A levels within diseased CF airways may create an environment for *P. aeruginosa* to safely repress the synthesis and assembly of the energy consuming and highly proinflammatory flagella [8]. In addition, the loss of flagellum will also decrease the chance of *P. aeruginosa* being phagocytized by SP-A-facilitated macrophage and neutrophils.

Our results suggest that the killing of flagellum-deficient mutants is more robust *in vivo* than *in vitro*. One likely explanation for the discrepancy is that our *in vitro* membrane permeability conditions do not accurately model the environment in the lung. It is also possible that other antimicrobial factors within alveolar space, including SP-A, SP-D, lactoferrin, β-defensins and other antimicrobial peptides and proteins [44], [45], collectively contribute to the killing of *P. aeruginosa in vivo*. We are currently examining whether these peptides act synergistically in clearance of *P. aeruginosa*.

In summary, our data suggest that *P. aeruginosa* flagella play an important role in resistance to pulmonary clearance by SP-A and in particular to SP-A-mediated membrane permeabilization. Therapeutic strategies aimed at blocking flagellar biosynthesis or formation, or aerosolizing SP-A or SP-A fragments into septic airways where SP-A levels are severely depleted, may be reasonable therapeutic approaches to explore in the future.

## Materials and Methods

### Reagents

All chemicals, except where noted, were obtained from Sigma Chemical Co. (St. Louis, MO).

### Bacterial strains, plasmids, media and growth conditions

All bacterial strains, plasmid vectors, and their derivatives are described in Table 1. Two *P. aeruginosa* PA01 strains [46], [47] and PA14 [48] were the wild-type strains used in the present study. The *P. aeruginosa* STM library [19] was created by random insertion of the transposon pUTmini-Tn*5* into the genome of PA01 strain from Holloway *et al* [46]. The PAO-*fliC* [27] and PAO-*fliD* [26] mutants, and the complemented *fliC* strain PAOC-fliC [49], and their isogenic parental PA01 (originally from Mike Vasil, see Table 1) [47] were the same as previously described. The *flgK*, δ*motAB*, δ*motCD* and δ*motAB*, δ*motCD* mutants, which were derived from PA14, was as previously described [29], [32]. Two mucoid clinical isolates of *P. aeruginosa*, CF51 and CF90, were obtained from the University of Cincinnati Adult Cystic Fibrosis Center following approval by the Institutional Review Board at the University of Cincinnati. *Escherichia coli K12* used as a control in some experiments was transfected with the plasmid PVL 1393 (Invitrogen, Carlsbad, CA) to confer ampicillin resistance. Plasmid pUCP19GFP (GFP^+^ Ap^r^), which constitutively expresses *gfp*, was used to label the strains for microscopic studies. Bacterial strains were grown in Luria broth (LB) for 16 hr at 37°C, and then suspended in LB with 20% glycerol and frozen in aliquots at −80°C. Before each assay, aliquots of the bacteria were cultured from frozen stocks in liquid LB or on LB agar plates (1.5% agar) with or without antibiotics for 3–16 hr at 37°C with continuous shaking, and then suspended in Tris-buffered saline, pH 7.4. Bacteria were cultured to stationary growth phase (OD600≈3.0) as indicated. The optical density at 600 nm was determined by spectrophotometry and correlated with numbers of viable bacteria by colony-forming units (cfu) after plating serial dilutions on agar plates. The antibiotic concentrations used were as follows: for *E. coli*, ampicillin (100 µg/ml); for *P. aeruginosa*, carbenicillin (300 µg/ml), gentamycin (100 µg/ml), kanamycin (100 µg/ml), rifampicin (50 µg/ml) and tetracycline (100 µg/ml).

### Purification of human SP-A

Human SP-A was purified from the lung washings of patients with alveolar proteinosis by a modification of the method of Suwabe et al, [50] and stored in 5 mM Tris, 150 mM NaC, pH 7.4, at −20°C. The preparations were deemed free of EDTA by a modified spectrophotometric assay of Kratochvil and White, using ß-phenanthrolene–disulfonic acid as the indicator [51].

### Protein assays

Routine protein concentrations were determined with a bicinchoninic acid protein assay kit (BCA; Pierce Chemical Co., Rockford, Illinois, USA) using BSA as a standard. Protein samples were separated on 8–16% SDS-PAGE gel and stained with Coomassie blue or silver.

### Animal husbandry

Swiss Black SP-A^−/−^ mice (a gift of J. Whitsett/T. Korfhagen) were developed from embryonic stem cells after disruption of the mouse SP-A gene by homologous recombination and maintained by breeding with Swiss Black mice, as previously reported [52]. The SP-A null allele was bred into the C3H/HeN background through nine generations, as described [15]. C3H/HeN control (SP-A^+/+^) mice were purchased from Charles River Laboratory (Boston). All comparisons made with the SP-A^−/−^ mice were with age and strain-matched C3H/HEN controls. All animals were housed in positively ventilated microisolator cages with automatic recirculating water located in a room with laminar, high efficiency particulate-filtered air. The animals received autoclaved food, water, and bedding. Mice were handled in accordance with approved protocols through the Institutional Animal Care and Use Committee at the University of Cincinnati School of Medicine.

### Comparative screening of the *P. aeruginosa* STM library in SP-A^+/+^ and SP-A^−/−^ mice

The *P. aeruginosa* PA01 STM library and screening methods were previously described [19]. Briefly, SP-A^+/+^ and SP-A^−/−^mice were anaesthetized using isofluorane and inoculated intranasally with 1×10^7^ cells of individual STM pools of 72 mutants in 50 µl of phosphate buffered saline buffer. After 16 hr of *in vivo* selection, lungs were removed from sacrificed mice, homogenized, and plated on LB agar. At least 10,000 bacterial colonies were harvested for multiplex PCR as described previously [17], [19]. Twenty sets of 72 mutants (total 1440) had been screened at the time of this writing. An additional 240 mutants previously shown to be attenuated in a chronic model of rat lung infection were also screened [19]. The mutants that were differentially cleared by SP-A during the *in vivo* passaging were subsequently retested by single PCR of bacterial cells recovered from three separate mice as previously described [17].

### Competitive and single infection assays *in vivo*

For competition assays, mice were inoculated intranasally with 1×10^7^ bacterial cells comprised of a 1:1 ratio of wild-type PA01 and *flgE*. Lungs were harvested 16 hr after infection for bacterial load determination. The competitive index (CI) is defined as the output ratio of mutant to wild-type bacteria divided by the input ratio of mutant to wild-type bacteria [21]. Thus, for a mutant strain that was less competitive than the parental strain from which it was derived, a CI value of <1 would be achieved. Single infections with 1×10^7^ *flgE* cells were performed by the intranasal route in SP-A^+/+^ and SP-A^−/−^ mice (group of five). Attenuation was defined as the log_10_ difference in CFU of *flgE* recovered from the lung tissues of SP-A^+/+^ and SP-A^−/−^ mice lung tissue 16 hr after inoculation.

### Alveolar macrophage and neutrophil phagocytosis assays *in vivo* and *in vitro*

For *in vivo* phagocytosis studies, 2.5×10^8^ GFP-expressing PA01 or *flgE* bacteria were intranasally inoculated into SP-A^+/+^ and SP-A^−/−^ mice. Two hours later, infected lungs were lavaged with PBS to obtain alveolar macrophages. For *in vitro* studies, freshly recovered mouse macrophages (∼5×10^5^ cells from 3 uninfected SPA^−/−^ mice) were adhered to chamber plastic culture slides, coated with 5% poly-D-lysine in 200 µl RPMI (Dulbecco's, containing 2.5 mg/liter gentamycin and 0.1% BSA), for 2 hr at 37°C in 5% CO_2_ and used immediately. Live GFP-expressing PA01 or *flgE* in RPMI were preincubated with or without 25 µg/ml human SP-A for 1 hr at 37°C with rotation. The media was removed from each well, replaced with 250 µl of opsonized PA01 or *flgE*, and incubated for 1 hr at 37°C in 5% CO_2_ at a ratio of 100 bacteria to 1 macrophage. Chamber slides were washed 3 times with PBS containing 1 mM CaCl_2_. Fluorescence from extracellular bacteria was quenched with crystal violet (0.8 mg/ml). Following two additional washes, cells were fixed with 1% paraformaldehyde in PBS plus 1 mM CaCl_2_ for 10 min, and stained with Evans blue for 2 min. The percentage of macrophages with engulfed bacteria was quantified by phase contrast fluorescence microscopy. At least 200 macrophages were counted. Human neutrophils were purified and cultured as previously described [53]. Neutrophil phagocytosis was performed as described for macrophages.

### Static growth experiments

Previous studies have demonstrated that mucoid strains of *P. aeruginosa* are capable of spontaneously reverting to a nonmucoid phenotype and acquiring flagellum based motility in a static growth conditions [28]. In this study, overnight cultures of two mucoid CF isolates of *P. aeruginosa* strains, CF51 and CF90, were inoculated into 5 ml LB in 18×150 mM culture tubes. These cultures were incubated at 37°C vertically in test tube racks without shaking for 72 hr, and then plated on regular LB agar. Individual nonmucoid colonies were replicated on swimming motility agar plates (LB solidified with 0.3% agar) to confirm reversion to motility phenotype.

### Flagellum-dependent swimming motility

Flagellum-dependent swimming motility was measured in fresh swimming motility agar plates with or without antibiotics. Plates were inoculated with 5 µl of an overnight culture of each strain of same cell density (as determined by spectrophotometer) and then incubated at 30°C for 24–48 hr. Motility was quantitatively assessed by examining the radius of opacity from the inoculation point, indicating the extent of bacterial swimming, compared to that of the wild-type PA01.

### *P. aeruginosa* membrane permeability assays

The effect of the SP-A or melittin on *P. aeruginosa* cell wall integrity was assessed by determining permeability to a phosphatase substrate, Enzyme-Labeled Fluorescence 97 (ELF-97, Molecular Probes) as described [17]. SP-A (50 µg/ml) or melittin (5 µg/ml) was incubated with 1×10^8^ logarithmic or stationary phase bacterial cells/ml in 100 µl of 5 mM Tris and 150 mM NaCl for 15 min at 37°C, and 100 µM ELF97 phosphatase substrate was added. Fluorescence was measured at excitation and emission wavelengths of 355 and 535 nm, respectively, for a period of 120 min.

### LIVE/DEAD bacterial staining

The viability of SP-A exposed bacteria was determined by LIVE/DEAD baclight™ Bacterial Viability Kit (Molecular Probes, Cat. No. L-7012). Briefly, stationary phase *E. coli* or *P. aeruginosa* bacteria were washed three times with 5 mM Tris, 150 mM NaCl, and adjusted to an OD_600 _of 1.0. A 100 µl aliquot of the cells was added to culture tubes containing SP-A (100 µg/ml) in 100 µl of 5mM Tris, 150mM NaCl, or to buffer alone as control. The mixtures were incubated at 37°C while shaking at 300 rpm for 1 hr, and stained according to the instructions provided by the supplier. The viability of the cells was checked under a fluorescence microscope. At least 1, 000 cells were counted.

### LPS purification and western blot analysis

*P. aeruginosa* PA01 and *flgE* cells were grown to late logarithmic or stationary phase in LB at 37°C and concentrated to A_560_ 1.5 in PBS, and verified to contain the same viable cell counts (data not shown). The resultant cell pellet from 500 µl of sample was resuspended in 100 µl of PBS and incubated with or without 100 µg/ml of SP-A at 37°C for 30 min. An equal volume of 2×sample buffer (6% SDS; 6% 2-mercaptoethanol; 10 mM dithiothreitol; 46% glycerol; 60 mM Tris, pH 8.0; 0.1% bromophenol blue) was added. The samples were boiled for 10 min and protein was digested by the addition of proteinase K to a final concentration of 50 µg/ml at 37°C overnight. The samples were boiled again for 10 min and cooled down to room temperature. A second volume of proteinase K, equal to the first, was added and incubated at 55°C for 3 h. Gross LPS profiles were determined by silver stain after SDS-PAGE electrophoresis.

Western blot analyses were performed as described [54]. Briefly, LPS samples (15 µl) were resolved by SDS-PAGE and electro-blotted to Immobilon P polyvinylidene difluoride membranes (Millipore, Bedford, MA). The membranes were then incubated for 60 min at room temperature in blocking solution (PBS containing 3% bovine serum albumin), followed by a 4-hr incubation with monoclonal antibodies against the inner core (MAb 7–4) and B-band O-antigen (MAb MF15-4) (generously provided by Dr. Joseph Lam, Guelph University), respectively. The membranes were hybridized with horseradish peroxidase-conjugated goat anti-mouse IgG secondary antibody. The immune complexes were visualized using the ECL Western Blotting Detection System (Amershan Biosciences, Piscataway, New Jersey) and Kodak BIOMAX (Kodak, Rochester, New York) X-ray films.

### Statistical analyses

Statistical analysis was performed using the Student's *t*-test and one-way analyses of variance (ANOVA). A significant difference was considered to be *p*<0.05.
